# Structure-Enhanced Stress Attenuation in Magnetically Tunable Microstructures: A Numerical Study of Engineered BCT Lattices

**DOI:** 10.3390/mi17010081

**Published:** 2026-01-07

**Authors:** Kuei-Ping Feng, Chin-Cheng Liang, Yan-Hom Li

**Affiliations:** 1 School of National Defense Science, Chung-Cheng Institute of Technology, National Defense University, Taoyuan 33551, Taiwan; z0912206565@gmail.com; 2 Department of Mechanical and Aerospace Engineering, Chung-Cheng Institute of Technology, National Defense University, Taoyuan 33551, Taiwan; a24618070@gmail.com

**Keywords:** magneto-rheological fluid, magnetic field, microbead chain, body-centered trigonal

## Abstract

Magnetorheological fluids (MRFs) exhibit dynamic, field-responsive mechanical properties, as they form chain-like and networked microstructures under magnetic stimuli. This study numerically investigates the structural and mechanical behavior of three-dimensional (3D) microbead chain assemblies, focusing on cubic and hexagonal body-centered tetragonal (BCT) configurations formed under compressive and magnetic field-driven aggregation. A finite element-based model simulates magnetostatic and stress evolution in solidified structures composed of up to 20 particle chains. The analysis evaluates magnetic flux distribution, total magnetic force, and time-resolved stress profiles under vertical loading. Results show that increasing chain density significantly enhances magnetic coupling and reduces peak stress, especially in hexagonal lattices, where early stress equilibration and superior lateral load distribution are observed. The hexagonal BCT structure exhibits superior resilience, lower stress concentrations, and faster dissipation under dynamic loads. These findings offer insights into designing energy-absorbing MRF-based materials for impact mitigation, adaptive damping, and protective microfluidic structures.

## 1. Introduction

Magnetorheological fluids (MRFs) represent a class of smart materials whose rheological properties can be rapidly and reversibly tuned under an external magnetic field. Composed of micron-sized magnetizable particles dispersed in a nonmagnetic carrier medium such as silicone or mineral oil, MRFs exhibit a remarkable liquid-to-solid transition known as the magnetorheological (MR) effect [1].

Their ability to dissipate mechanical energy efficiently under controllable magnetic fields has made them highly valuable in various engineering applications [2,3,4], including brake systems [5], controlled vibration dampers [6,7] and sealing mechanisms [8], because of their unique rheological characteristics. These rheological properties are influenced by numerous factors, which are classified as either intrinsic or extrinsic, based on their origin. Intrinsic factors include particle shape, size distribution and carrier fluid viscosity [9,10]; extrinsic factors include magnetic field strength, shear rate, temperature, and compression [11,12]. The interaction between these factors is used to develop strategies to enhance MR fluid performance in practical applications. MRFs are assessed in terms of micromechanical properties, including flow behavior, yield stress, and viscoelastic responses under shearing flows. Of particular significance is yield stress, which is the minimum stress required to initiate flow. It is directly proportional to the force required to disrupt the chain-like structures that form under the influence of an external magnetic field. The chain-like or column-like structures are enhanced as the magnetic force is increased in a strong magnetic field [13,14].

To achieve adaptive energy absorption and enhance the energy absorption capacity of porous materials, the unique rheological characteristics of MRFs were applied to design a novel energy-absorbing material [15,16,17]. Additionally, experimental studies were conducted on the energy absorption characteristics of lattice structures, foam materials, and honeycomb structures filled with shear-thickening fluids [18,19,20]. However, there is still a lack of reported information on the impact behaviors and energy absorption of porous materials filled with intelligent fluids.

The macroscopic properties of MRFs depend on their interior particle-formed microstructures [21,22]. As high-performance MRFs continue to evolve to meet the demands of various operational environments, there is a need to gain a deeper understanding of the intricate microstructures within MRFs. However, the feasibility of studying their microscopic attributes is profoundly limited by experimental techniques and conditions. Conventional methods, such as scanning electron microscopy (SEM), light microscopy, and X-ray diffraction, are primarily used to analyze internal structures and determine the material phase composition; however, these methods entail sample disruption, so subsequent assessments of the mechanical properties of MRFs and comprehensive 3D structural analysis are impossible.

Despite significant progress, understanding the mechanical characteristics of 3D particle chain assemblies under field-induced solidification remains limited. Traditional MRF studies focus on bulk rheology or single-chain behaviors, whereas the collective response of multi-chain, three-dimensional structures under external loading and magnetic coupling has received less attention. Such knowledge is crucial for developing next-generation magnetically tunable porous materials capable of adaptive energy dissipation in smart structures, impact absorbers, and microfluidic energy devices.

While spontaneous self-assembly in MR fluids often results in disordered chains or “jammed” aggregates due to kinetic trapping, previous research has demonstrated that ordered structures can be engineered [23,24]. Specifically, a compression-assisted aggregation method is experimentally developed by applying compressive strain along the direction of the magnetic field. As a result, the fluid is forced to transition from disordered chains into robust, thick columns. These columns exhibit a body-centered tetragonal (BCT) lattice structure, which is the energetic ground state of magnetized spheres. Consequently, the 3D microstructures modeled in this study are not merely theoretical ideals but represent this specific, structure-enhanced state of MR fluids, where yield stresses are maximized.

In this study, we perform a numerical investigation of 3D solid structures composed of multiple microbead chains subjected to an external magnetic field. By systematically varying the number and arrangement of particle chains, we evaluate the influence of geometric configurations on magnetic flux distribution, total magnetic force, and stress responses. While in a 1D chain, only one possible configuration can be obtained, 2D and 3D structures can be arranged in a cubic or hexagonal pattern. The effect of different arrangement configurations on the total magnetic force within these structures is determined. The study also determines the load-bearing capacity and stress distribution uniformity in particle-formed microstructures with various arrangements by applying a vertical compressive force. The study findings offer insights into designing energy-absorbing MRF-based materials for impact mitigation, adaptive damping, and protective microfluidic structures.

## 2. Simulation Model and Geometry

### 2.1. Parameters of Magnetic Beads

**Figure 1:**
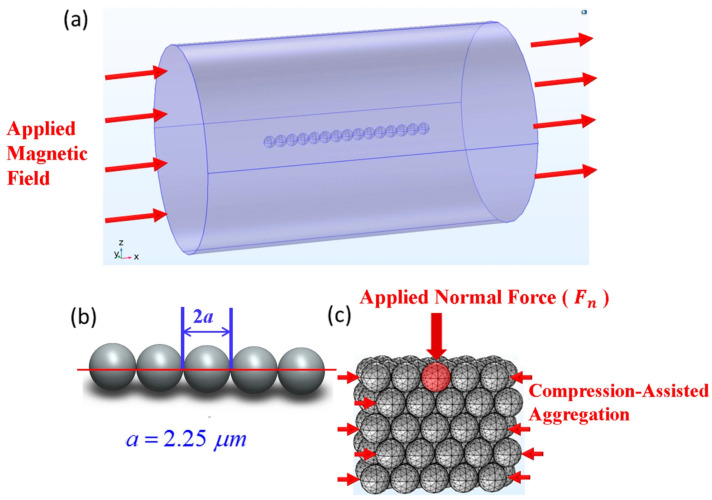
Computational model and geometric definition of the physical MR fluid structures. (**a**) Geometry of the numerical domain acting as a Representative Volume Element (RVE), where a single particle chain is immersed in the carrier fluid with magnetic insulation boundaries to simulate an isolated control volume. (**b**) Definition of the evaluation axis (central line) aligned with the external magnetic field vector, used to calculate the interparticle magnetic flux density and stress transmission along the chain. (**c**) Discretized 3D finite element mesh of the body-centered tetragonal (BCT) lattice. This mesh topology is physically derived from the “super-strong” columnar structures formed via the longitudinal compression technique, Refs. [23,24], allowing for the simulation of the stress distribution when a vertical compressive force is applied to the engineered aggregate.

When a magnetic particle with a radius a is subjected to a uniform external magnetic field *H*, the magnetic moment m of this particle is [21,25]

(1) $$m=\frac{4}{3}\pi a^{3}M$$

where *M* represents magnetization and *M* = *χH*, *χ* is the magnetic susceptibility of the particle. The relationship between the magnetization *M* and magnetic field *H* with a magnetic flux density *B* is written as

(2) $$B=\mu_{0}(H+M)=(1+\chi )H=\mu_{0}\mu_{r}H=\mu H$$

where *μ*_0_, *μ_r_*_,_ and *μ* are the permeability of a vacuum, the relative permeability, and the absolute permeability, respectively. In a magnetic particle chain, the magnetic force on a ferromagnetic particle also strongly depends on the other magnetized particles. Thus, the total magnet force can further be separated into two parts, namely the magnetic force induced by the gradient magnetic field generated by the external field, and the induced magnetic force, which is induced by the additional magnetic field generated by the other magnetized particles in the chain. When a chain composed of *N* particles is exposed to an external field with strength *H*, the vector quantity of total magnetic force *F_m_*, which includes additional magnetic fields that are produced by other particles, is calculated using the enhanced dipole model, which is written as [25,26]

(3) $$F_{m}=\sum_{\begin{array}{l} i=1\\ j\neq i \end{array}}^{N}\frac{4\pi \mu_{0}a^{6}H^{2}\chi^{2}}{3d_{ij}^{5}}\left\{\left[\left(1-5\cos^{2}\theta )-\frac{a^{3}\chi }{3d_{ij}^{3}}(1+4\cos^{2}\theta \right)\right]{\vec{r}}_{ij}+2d_{ij}\cos\theta (1+\frac{a^{3}\chi }{6d_{ij}^{3}})\vec{k}\right\}$$

where *d_ij_* is the distance from particle *i* to particle *j*, ${\vec{r}}_{ij}$ is the relative position vector from particle *i* to particle *j*, *θ* is the angle between the center connecting line for two particles and the external magnetic field, and $\vec{k}$ is the unit vector for the external magnetic field. All the governing equations are numerically solved using COMSOL Multiphysics 6.2. Figure 1 shows the geometry and grid structure of the model that simulates the magnetic flux density and the stress distribution for various 3D structures that are composed of multiple magnetic microbead chains.

The micro-sized superparamagnetic particles for this study are composed of iron oxide magnetite (Fe_3_O_4_) embedded in a polystyrene microsphere. Assuming the particles in a chain are identical spheres and a single Fe_3_O_4_-filled polystyrene microsphere corresponds to a single magnetic microbead, the properties of the commercial available microbeads (Dynabeads M-450 EpoxyThermo Fisher Scientific Baltics UAB, Vilnius, Lithuania) are as follows: density ρ = 1500 kg/m^3^, radius *a* = 2.25 μm with initial magnetic susceptibility *χ* = 1.6, saturation magnetization *Ms* = 350–400 Oe, and an absence of magnetic hysteresis. The nominal loading (volume fraction) of the MR fluid is 32 vol% iron oxide equivalent. The strength of the external magnetic field is 5440 A/m, which is an overall moderate field above the threshold for chain formation without rupture (N*Mason number^0.5^ < 1.7) but below the full saturation shown in a previous study [27]. Each particle chain in the model is initialized with beads in full contact (center-to-center distance = 2a). This reflects the state after magnetic attraction (and any applied compression) has pulled particles together.

The geometric configurations analyzed for the stress distribution in this study, shown in Figure 1c, are based on the body-centered tetragonal (BCT) lattice structure. This specific packing is chosen to simulate the internal structure of the “super-strong” columns formed via the longitudinal compression technique [23,24], which eliminates voids and defects typical of spontaneous assembly.

### 2.2. Simulation Setup, Mesh Independence, and Validation

The numerical model was solved using the COMSOL Multiphysics with an iterative solver (relative tolerance set to 1 × 10^−6^). To simulate an isolated control volume of the MR fluid, the computational domain was defined as a block of carrier fluid surrounding the microbead chains. Magnetic insulation boundary conditions (n·B = 0) were applied to the external surfaces of the fluid domain. To ensure that the boundary proximity did not artificially constrict the magnetic field lines (domain sensitivity), the domain boundaries were extended to a distance of at least 10 times the particle radius from the outermost beads. Magnetic flux continuity was applied at all bead–fluid interfaces.

To ensure numerical stability, a mesh independence study was conducted on a representative 20-chain structure with a body-centered tetragonal (BCT) lattice. Three different mesh densities were evaluated, corresponding to element counts of 84,721 (Case 1), 93,382 (Case 2), and 106,149 (Case 3). We monitored the total normal stress acting on the structure as the convergence metric. As shown in Table 1, the variation in the calculated stress between Cases 2 and 3 was found to be approximately 0.92%. Consequently, the mesh settings for Case 2 (93,382 elements) were selected for all subsequent simulations to balance computational efficiency with high numerical accuracy.

Furthermore, to validate the accuracy of our magnetic parameters (specifically magnetic susceptibility *χ* and saturation magnetization Ms), we compared our numerical results with the established experimental and analytical data provided in Ref. [27] under identical magnetic field conditions. We simulated single chains consisting of 13 and 15 microbeads (denoted as P13 and P15, respectively) under varying magnetic field strengths and calculated the maximum normal force between adjacent particles. The comparison revealed excellent agreement with the results shown in Figure 2 of Ref. [27], with the relative variation ranging between only 0.21% and 0.39%. This low deviation confirms the validity of our governing equations and parameter selection for predicting interparticle magnetic forces.

**Figure 2:**
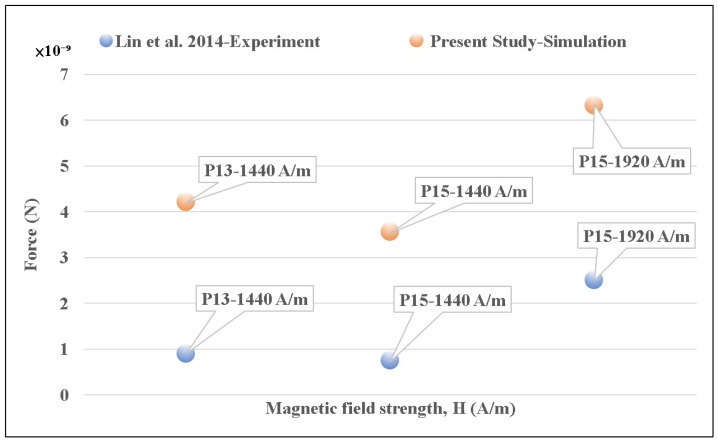
Numerical validation of magnetic parameters: comparison of the calculated maximum normal force between adjacent microbeads in single chains (P13 and P15 denoted as chains composed of 13 and 15 particles, respectively) against the established experimental data from Ref. [27].

## 3. Results and Discussion

### 3.1. Effect of Microbead Arrangement on Magnetic Field Intensity of Particle Chain Structure

**Figure 3:**
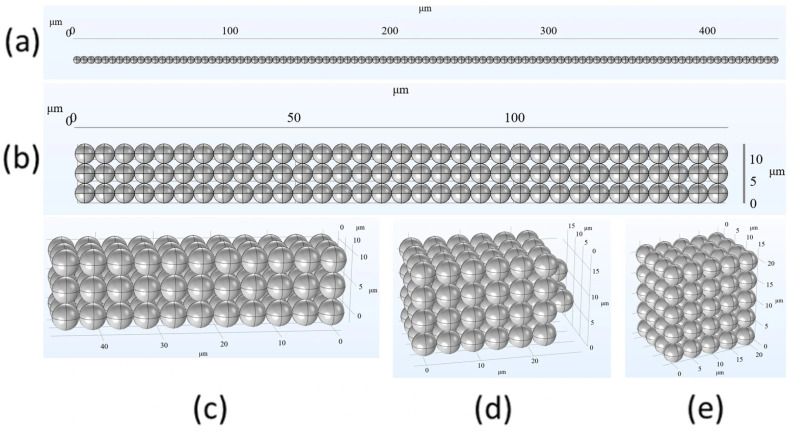
Schematic diagrams of 99 MRF magnetic particles that are arranged in the following configurations: (**a**) single chain; (**b**) three chains; (**c**) nine chains; (**d**) sixteen chains; (**e**) twenty chains.

A dimensionless Mason number [28,29], which is the ratio of the viscous effect to the magnetic effect, indicates that a stronger field strength increases the structural stability of the particle chains. The magnetic forces (*F_m_*) between two individual beads, which is determined by the magnetization (*M*) and external field (*H*), significantly affect the structural stability of particle chain structures [27]. The variety of arrangements and the length of particle chains result in different strengths for the structures. This study uses various combinations of microbeads, including 1D, 2D, and 3D solid-like structures, as shown in Figure 3. In a 1D chain, only one possible configuration can be obtained, while 2D and 3D structures can be arranged in a cubic or hexagonal pattern. The 1D structure consists of a single chain of 99 particles (1 × 99). The 2D structure consists of three chains, each of which has 33 particles (3 × 33). The 3D structure is divided into 9 chains of 11 particles (9 × 11), 13 chains of 6 particles plus 3 chains of 7 particles (13 × 6 + 3 × 7), and 19 chains of 5 particles plus 1 chain of 4 particles (19 × 5 + 1 × 4). Each computational model consists of 99 particles in total. This is used to simulate the difference in the total magnetic field intensity and structural strength for the same number of particles with different combinations.

**Figure 4:**
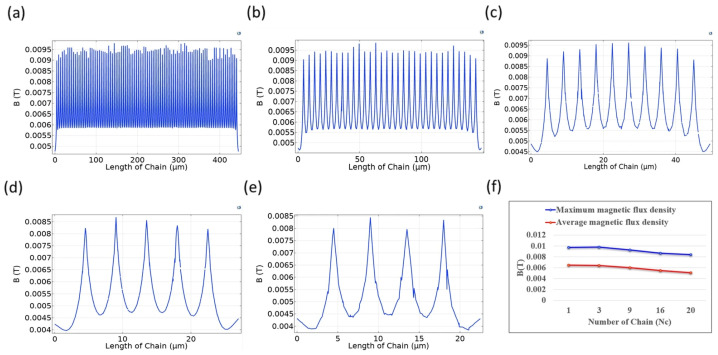
Magnetic field intensity distribution for 99 magnetic particles in various arrangements: (**a**) single chain; (**b**) three chains; (**c**) nine chains; (**d**) sixteen chains; (**e**) twenty chains; (**f**) a comparison of the maximum and average magnetic flux density for cases (**a**–**e**).

Figure 4 illustrates the magnetic field intensity distribution between magnetic particles in various arrangements, as calculated using COMSOL Multiphysics 6.2 for the configurations depicted in Figure 3. Figure 4a shows the magnetic flux density distribution between 33 magnetic particles in the central region of a single chain composed of 99 magnetic particles. The magnetic field intensity distribution is uniform and there are no significant variations in magnetic flux density between individual particles. The maximum value is approximately 0.01 T. If the length of the single chain is reduced to 33 particles, as shown in Figure 4b, the maximum magnetic flux density is nearly the same as the case shown in Figure 4a. Furthermore, the magnetic field intensity distribution in the central region remains uniform, but there is a slight decrease in the magnetic flux density between particles near both ends.

Figure 4c–e show that as the number of particles in the single chain decreases, the uniformity of the magnetic field intensity between particles significantly decreases and the maximum magnetic flux density decreases. Figure 4e shows that the maximum magnetic flux density is 0.0085 T, which is approximately 10% lower than the maximum magnetic field intensity in Figure 4a.

Figure 4f shows a comparison of the maximum and average magnetic flux density for the single chains in Figure 4a–e. Longer chains exhibit a more uniform magnetic flux density distribution and greater field intensity. The reason for this is that for longer chains, the effect of additional magnetic fields that are produced by other particles in a single chain is more significant. The magnetic field lines between particles at both ends also form closed loops from the north pole to the south pole, so the magnetic field lines are not distributed in a straight-line manner and a weaker magnetic flux density is measured near the endpoints than at the central position. The shorter the chain, the more pronounced this effect is.

### 3.2. Magnetic Force and Structural Stability of Particle Chain Assemblies

**Figure 5:**
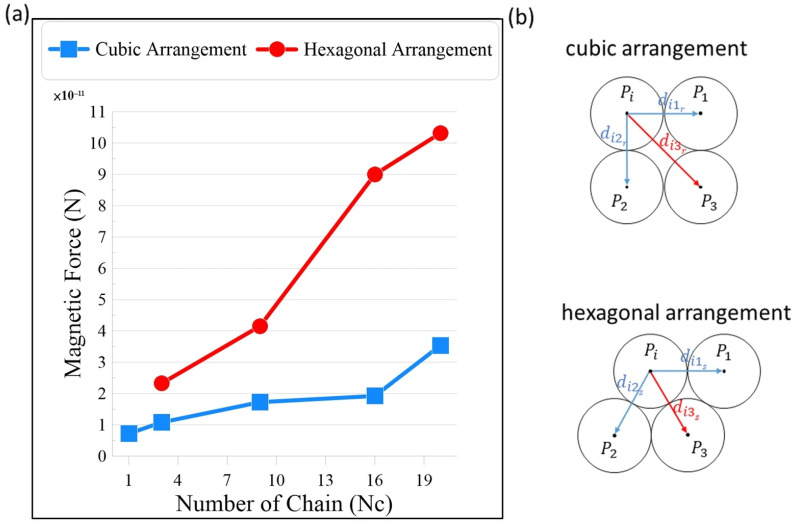
(**a**) Comparison of the magnetic force on particle chains for different arrangements with a solid-like structure; (**b**) a schematic diagram of the mutual induction mechanism between magnetic particles in a cubic and hexagonal arrangement.

The total magnetic force generated within the particle chain structure directly influences the binding force between neighboring microbeads and consequently governs the structural stability of the magnetically induced 3D assembly. Figure 5a presents the relationship between the total magnetic force and the number of chains for both cubic and hexagonal configurations. In both arrangements, the magnetic force increases almost linearly with the number of chains, reflecting the cumulative contribution of inter-particle magnetic interactions as additional chains are introduced. This trend indicates that particle network expansion enhances the overall field-induced cohesion within the microstructure, thereby improving its resistance to external loading.

A closer comparison reveals that the hexagonal arrangement consistently exhibits slightly higher total magnetic force than the cubic configuration for the same number of chains. This enhancement arises from the more efficient magnetic flux linkage between adjacent particles in the hexagonal lattice, where each particle has more neighboring interaction paths and shorter magnetic distances. As illustrated in Figure 5b, the mutual induction mechanism between particles differs notably between the two configurations. In the cubic arrangement, the magnetic induction primarily occurs along orthogonal directions (e.g., between particles Pi–P1 and Pi–P2), resulting in weaker lateral coupling. In contrast, the hexagonal structure forms triangular magnetic loops between adjacent particles (Pi–P1-P2–P3), which strengthen the local magnetic field intensity and promote more uniform magnetization across the structure.

The stronger magnetic connectivity in the hexagonal arrangement enhances inter-chain binding and contributes to greater structural rigidity and stability in the aggregate. This effect on stress resistance and energy absorption will be discussed in the following sections.

### 3.3. Stress Distribution in Aligned Particle Chains

This section analyzes the mechanical performance and theoretical magnetic yield stress of the proposed 3D microbead chain structures under an applied magnetic field.

In this analysis, the microbeads are modeled as rigid bodies. This assumption is chosen to isolate the magnetic contribution to the structural lattice strength, independent of polymer matrix compliance. The validity of assuming a stable, non-ruptured single chain under load is justified by the experimental stability criteria established in Ref. [27]. Their work demonstrated that superparamagnetic bead chains maintain structural integrity and do not rupture when the applied magnetic field intensity exceeds a critical threshold of approximately 1922 A/m (corresponding to an interparticle magnetic force of ~3 × 10^−9^ N, resulting in N*Mason number ^0.5^ < 1.7). In our simulation, the applied magnetic field is 5440 A/m, which significantly exceeds this critical stability threshold. Consequently, the magnetic clamping forces are sufficient to suppress chain rupture within the engineered BCT structure, making the rigid body assumption valid for determining the theoretical magnetic yield limit of the lattice topology.

**Figure 6:**
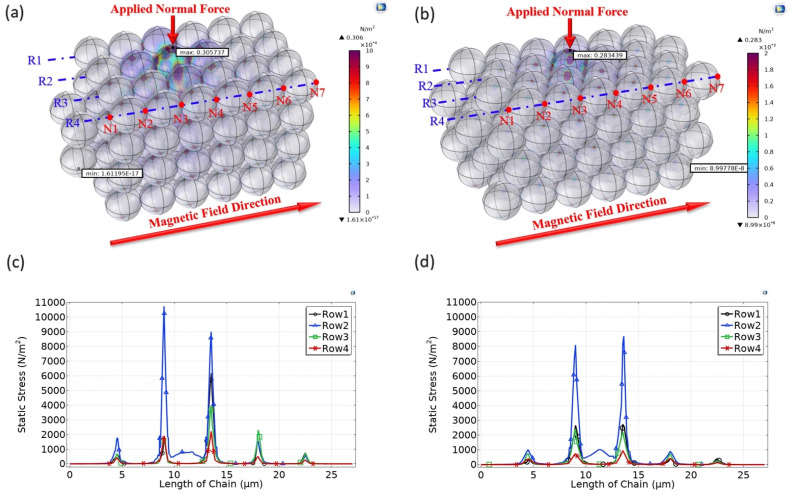
Contour map of stress distribution for a 3D structure consisting of 16 chains in (**a**) a cubic and (**b**) hexagonal arrangement, and (**c**,**d**) the comparison of the stress distributions on each node in a chain on different rows for the first layer of the 3D structure in (**a**,**b**), respectively.

Figure 6 presents the stress distribution for the simple cubic and hexagonal packing configurations, composed of 99 magnetic particles arranged in 16 chains, under an applied field of 5440 A/m and a vertical compressive force of 6.3 × 10^−9^ N. As expected due to the point contact nature of the rigid spheres, high stress concentrations are observed precisely at the interparticle contact points in both configurations where magnetic attraction is highest. However, a comparison reveals distinct differences in stress management. The hexagonal packing (Figure 6b) exhibits a markedly more uniform stress distribution across the lattice compared to the localized high-stress zones in the simple cubic packing (Figure 6a).

Quantitatively, the average maximum stress recorded in the critical regions of the simple cubic structure is approximately 10,000 N/m^2^, whereas the hexagonal structure shows a lower average maximum stress of approximately 8500 N/m^2^. This represents a stress reduction of approximately 15% in the hexagonal configuration. This significant reduction is directly attributed to the higher coordination number in the hexagonal packing (where internal beads have up to 12 contact neighbors, compared to 8 in the cubic arrangement). This increased connectivity allows for more efficient magnetic flux distribution and load transfer throughout the lattice. Consequently, the hexagonal structure (representing the cross-section of BCT columns) demonstrates superior static load-bearing capacity, indicating its potential for higher performance in energy absorption applications compared to simple cubic arrangements.

### 3.4. Dynamic Load Redistribution in Varying Chain Densities

**Figure 7:**
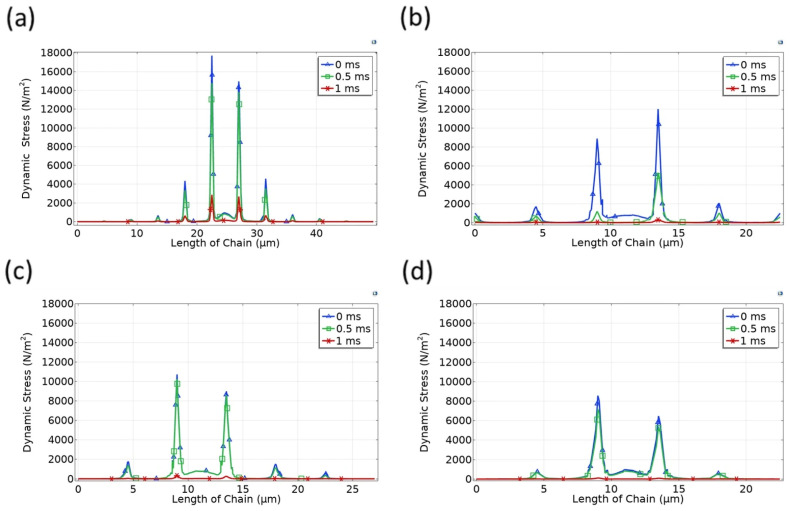
Time-dependent stress distribution on the top layer of cubic microbead chain structures under vertical compression: (**a**) 3-chain structure; (**b**) 9-chain structure; (**c**) 16-chain structure; (**d**) 20-chain structure.

To evaluate the dynamic load-bearing capability of magnetically aligned microstructures, we analyzed the time evolution of stress along the top chain layer under vertical compressive loading. Figure 7 displays the dynamic load-bearing capacity of aligned particle chains arranged in cubic configurations with 3, 9, 16, and 20 chains at different time points (0 ms, 0.5 ms, and 1 ms).

At the initial moment of loading (t = 0 ms), the stress is sharply concentrated in the central chains for all configurations. Notably, the maximum stress in the three-chain arrangement (Figure 7a) reaches nearly 18,000 N/m^2^, indicating intense localized force transmission. As the number of chains increases to 20 (Figure 7d), the maximum stress is reduced to ~8500 N/m^2^, demonstrating a decrease of around 53% in peak stress. This significant reduction highlights the role of lateral chains in immediately redistributing the applied load, consistent with the mechanical characteristics of denser BCT-like structures.

As time progresses to t = 1 ms, the stress profile further evolves. Across all configurations, there is a noticeable broadening of the stress distribution and a decrease in peak stress values, particularly in the 16- and 20-chain cases (Figure 7c,d). The 20-chain configuration exhibits the most uniform and lowest stress magnitudes across the top layer, suggesting that higher lateral chain density leads to greater dynamic resilience. This behavior is attributed to enhanced lateral force transmission and the formation of staggered magnetic chain networks that support stress spreading in three dimensions.

**Figure 8:**
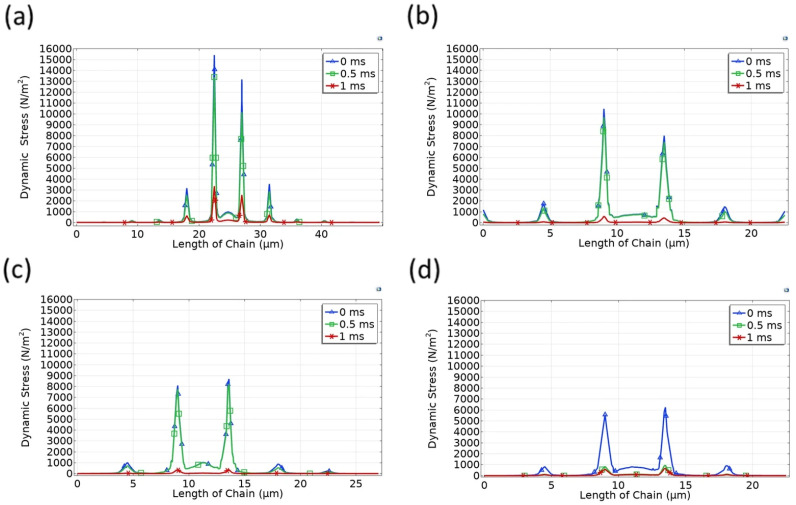
Time-dependent stress distribution on the top layer of hexagonal microbead chain structures under vertical compression: (**a**) 3-chain structure; (**b**) 9-chain structure; (**c**) 16-chain structure; (**d**) 20-chain structure.

In addition to cubic configurations, we examined the hexagonal microbead chain arrangements to assess how the packing geometry influences dynamic stress responses under compressive loading. Figure 8 presents the time-dependent stress distribution for 3-, 9-, 16-, and 20-chain hexagonal arrangements at 0 ms, 0.5 ms, and 1 ms.

Compared to the cubic configurations, all hexagonal setups demonstrate reduced initial peak stress under the same loading conditions. For instance, the maximum stress for the three-chain hexagonal case is around 15,000 N/m^2^, while the 20-chain structure starts at approximately 6000 N/m^2^—both lower than their cubic counterparts. This suggests that hexagonal chain packings may better accommodate the imposed normal stress due to more uniform contact angles and stronger lateral interactions.

As the simulation evolves to t = 1 ms, the stress in all configurations drops considerably. Notably, in the 20-chain hexagonal structure (Figure 8d), the stress distribution reaches a nearly uniform and minimized state as early as 0.5 ms, indicating faster stress relaxation compared to the same-density cubic structure. This early equilibrium implies superior shock dissipation capability in hexagonal arrangements, possibly due to the enhanced coordination between adjacent chains in a close-packed pattern.

**Figure 9:**
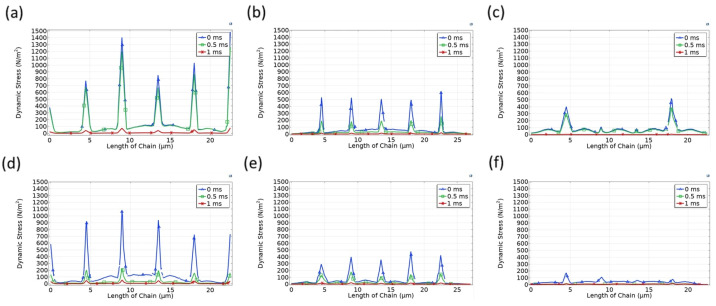
Time evolution of stress distribution along the bottom row of microbead chains under vertical compression for (**a**–**c**) cubic and (**d**–**f**) hexagonal arrangements with 9, 16, and 20 chains, respectively.

To further investigate how mechanical stress distributes and dissipates through the depth of the microbead assemblies, we examined the stress evolution on the bottom row of 9-, 16-, and 20-chain structures in both cubic and hexagonal arrangements, as shown in Figure 9.

Across all cases, a consistent trend emerges: stress transmitted to the bottom row is significantly attenuated, particularly as time progresses. At t = 0 ms, moderate levels of stress are detected, but these diminish rapidly over the subsequent time frames. By t = 1 ms, the stress levels in the bottom row are nearly negligible for both geometries and all packing densities, indicating that the majority of the impact load has been absorbed or redistributed in the upper and intermediate layers.

## 4. Conclusions

This study presents a comprehensive numerical evaluation of 3D microbead chain assemblies inspired by body-centered tetragonal (BCT) lattice structures, mimicking experimentally realizable solidified states of MRFs under compressive strain. Simulations confirm the following:

Magnetic enhancement: Increasing the number of chains enhances total magnetic force and internal cohesion, especially in hexagonal packing due to stronger lateral coupling and denser magnetic flux linkage.

Stress mitigation: Higher packing densities reduce peak stress values by over 50% and promote uniform stress redistribution, improving both static and dynamic load-bearing performance.

Geometry effects: Hexagonal arrangements consistently outperform cubic ones in stress damping and equilibration rate. In particular, the 20-chain hexagonal structure exhibits near-complete stress homogenization within 0.5 ms, demonstrating rapid response to impact.

Bottom-layer isolation: Time-dependent analysis reveals that stress is effectively dissipated in the upper layers, with negligible transmission to the bottom row, a desirable trait for stress isolation in layered MRF devices.

Overall, hexagonally packed BCT-inspired structures provide an optimal architecture for tunable stress absorption in MRF-based materials. Future work will incorporate non-rigid microbead deformation, explore the effect of magnetic field strength and bead size, and perform experimental validation of the BCT columns to extend applicability in real-world adaptive damping systems.

## Figures and Tables

**Table 1:** Mesh independence study performed on a representative 20-chain structure. The total stress was evaluated across three different mesh densities.

Mesh	Case 1	Case 2	Case 3
Total Mesh	84,721	93,382	106,149
Maximum element size (μ m)	1.36	1.33	1.3
Minimum element size (μ m)	0.099	0.077	0.043
Stress (N/m^2^ )	6211.7	6242.1	6300.3
Variation (%)	1.41%	0.92%	N/A

## Data Availability

The raw data supporting the conclusions of this article will be made available by the authors on request.
